# Congenital posterior urethral diverticula causing bladder outlet obstruction in a young male

**DOI:** 10.4103/0970-1591.42630

**Published:** 2008

**Authors:** Saurabh Agrawal, M. S. A. Ansari, R. Kapoor, D. Dubey

**Affiliations:** Department of Urology and Renal Transplantation, Sanjay Gandhi Post Graduate Institute of Medical Sciences, Lucknow - 226 014, UP, India

**Keywords:** Bladder outlet, diverticulae, urethra

## Abstract

We present a case of 26-year-old male presenting with mild renal failure. Ultrasound findings were suggestive of posterior urethral valve, but micturating cystourethrogram and endoscopic evaluation confirmed the diagnosis of posterior urethral diverticulae. Transurethral resection of diverticulae was performed. Patient is voiding well and his renal function has stabilized.

## INTRODUCTION

While the adult presentation of posterior urethral valve (PUV) causing bladder outlet obstruction with back pressure changes is known, there are only anecdotal reports of congenital posterior urethral diverticula presenting in a similar fashion.[1] We describe the case of an adult male presenting with obstructive uropathy with congenital posterior urethral diverticula.

## CASE REPORT

**Figure 1:**
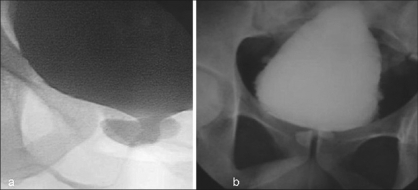
(a) MCU showing dilated posterior urethra with filled up diverticula causing circumferential compression from behind. Note the sharp cut-off of voided contrast at the level of prostatic urethra. (b) Postvoid MCU film showing filled up diverticula causing urethral obstruction with large residual urine in bladder

**Figure 2:**
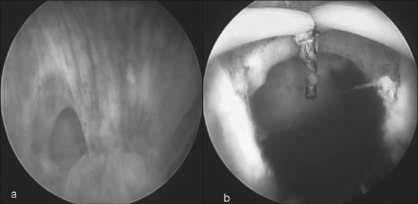
(a) Cystoscopic view: note the right paramedian ostium of posterior urethral diverticula just above the verumontanum. (b) After transurethral marsupilization - creating a wide channel communicating with bladder and urethra

A 26-year-old male presented with complaints of straining to void, postvoid dribbling and sensation of incomplete emptying of urine with nocturnal enuresis since as long as he could remember, but he was never concerned about it. He was being evaluated for chest infection and was incidentally diagnosed to have deranged renal function and was thus further evaluated. Physical examination was unremarkable. Laboratory evaluation revealed normal hemogram, BUN 93, serum creatinine 2.5 mg/dl. Microscopic examination of urine revealed numerous pus cells and urine culture positive for *Klebsiella*. Ultrasound revealed moderate hydroureteronephrosis with thickened, trabeculated bladder and dilated posterior urethra. The patient was stabilized with a trial of per urethral catheter and proper antibiotic cover (S. creatinine came down to 1.5 mg/dl and urosepsis was controlled). Micturating cystourethrogram (MCU) revealed cystic dilatation in the region of posterior urethra compressing it from behind resulting in obstruction with large postvoid residual urine volume [Figure 1]. Pressure flow studies were suggestive of poorly compliant bladder with a residual urinary volume of 320 ml. Cystourethroscopy revealed a right paramedian, pus discharging ostium in the region of posterior urethra proximal to verumontanum. Further inspection after dilatation of ostium revealed an epithelized cavity full of infective material with normal surrounding epithelium suggestive of a diverticula. Transurethral marsupilization of diverticula with Collin's knife was performed [Figure 2]. Patient is voiding with good flow with postvoid residual urine of 50 ml only. At one-year follow up, he was relieved of his lower urinary tract symptoms with stable renal function (S. Creatinine - 1.3 mg/dl).

## DISCUSSION

Diverticula in urethra may be defined as saccular evaginations of urethral mucosae. In contrast to anterior urethral diverticula, which are primarily congenital, posterior urethral diverticula are generally of acquired origin. In a review including 95 diverticulae of posterior urethra, only six were of congenital origin.[2] Multiparous females with urethral trauma from childbirth are clearly predisposed. Other causes include external or surgical trauma, catheterization, periurethral (particularly prostatic) suppurative infections, calculi and distal obstruction.[2] Faulty/incomplete fusion of a segment of the urethral plate is the proposed mechanisms for the congenital variety. In this case, absence of a predisposing cause with chronic backpressure changes on imaging suggests a congenital etiology.

Among differential diagnosis of cystic lesions of prostate include Mullerian duct cysts and utricular cysts. Both are midline cyst with opening into verumontanum,[3] whereas the diverticula in this case communicated with the urethra proximal to verumontanum and in a paramedian position. Uncommonly ejaculatory and vas deferens cyst, prostatic retention cyst, posterior bladder diverticulum present in a similar manner. Though congenital urethral diverticula are seen in early teens, late presentation - as in this case, though rare, can be seen. Presentation of posterior urethral diverticula can be diverse, ranging from asymptomatic lesion diagnosed incidentally on endoscopy to symptomatic presentation with postvoid dribbling or urinary tract infection (frequency, urgency, dysuria, pyuria, tenesmus and perineal pain). It might be further complicated with calculi, infection and carcinoma (adenocarcinoma). Proposed etiopathogenesis is that it can swell up significantly during micturition compressing bladder neck and urethra from posterior aspect resulting in outlet obstruction and its antecedent complications.[4]

Imaging studies help in not only characterizing the lesion, but also evaluating the antecedent complications. This includes not only a baseline ultrasound, but also MCU to look for reflux, bladder neck opening and characteristic filling of the diverticula. Postvoid films provide an insight into the severity of the lesion as in this case, which revealed incomplete emptying of both diverticula and trabeculated bladder. Urodynamic studies should be performed to characterize bladder and urethral physiological behavior. Findings of silent electromyographic during voiding went against dysfunctional voiding. Not until we had taken up the case for cystourethroscopy, which revealed a right paramedian ostium leading into an epithelized cavity full of infective material, with normal surrounding epithelium, could we reach to a definitive diagnosis of posterior urethral diverticula. Simultaneous rectal compression to empty the diverticular contents gave indirect evidence to the diagnosis.

Initial management of such patients is online with PUV - placement of per-urethral catheter, stabilization of electrolyte imbalance, control of infection and normalization of renal function status. Definitive management can be both endoscopic and open surgical with diverticular excision and patch graft urethroplasty.[5] In this case, endoscopic marsupilization of diverticula was carried out to create a wide communicating channel between bladder and diverticula and urethra, thus ensuring proper emptying of diverticula [Figure 2].

A high index of suspicion of posterior urethral diverticulum should be kept in mind while evaluating a young patient of lower urinary tract symptoms. Systematic imaging and other studies should be performed to corrobrate with the clinical findings and to enable to reach to a definitive diagnosis. Early recognition and prompt treatment can prevent antecedent complications associated with this entity and preserves renal function.
